# Case Study: The Surgical Management of Angiokeratoma Resulting from Radiotherapy for Penile Cancer

**DOI:** 10.1100/tsw.2009.23

**Published:** 2009-05-20

**Authors:** Rowland Rees, Alex Freeman, Peter Malone, Giulio Garaffa, Asif Muneer, Suks Minhas

**Affiliations:** ^1^ Institute of Urology, University College London Hospital, London, United Kingdom; ^2^ Royal Berkshire Hospital, Reading, UK

**Keywords:** angiokeratoma, radiotherapy, penile cancer, VRAM flap, skin graft

## Abstract

Angiokeratoma is a rare, benign skin lesion and a recognised complication of radiation therapy. Here we describe a case of extensive angiokeratoma of the groin and external genitalia resulting from external beam radiation to that area in a patient with penile carcinoma. Furthermore, we outline the management of this problem by surgical reconstruction.

## CASE REPORT

A 62-year-old gentleman presented with a 3-month history of painful, blistering, and discharging lesions involving the skin of his right groin, penis, and right hemiscrotum. He also had significant scrotal lymphoedema and a buried penis (Fig. 1). Three years previously, he had undergone a glansectomy and right inguinal lymphadenectomy for a T2 G2 N1 penile carcinoma. This was followed by 50 Gy of external beam radiotherapy to the groin and genital region due to extranodal spread of disease. There had been no disease recurrence, but the skin condition caused a significant deterioration in quality of life due to pain and discharge. The affected area corresponded to the field of irradiation and biopsies revealed angiokeratoma, with no evidence of malignancy. A CT scan also revealed no evidence of recurrent disease.

Following discussion, the patient underwent *en bloc* excision of the affected area of skin in the groin, penis, and scrotum, with simultaneous scrotectomy for the scrotal lymphoedema. The lateral scrotal skin flaps were closed primarily over the testes, the penis mobilised and grafted with a partial-thickness skin graft. The groin defect was replaced with a rotation Vertical Rectus Abdominus Myocutaneous (VRAM) flap (Fig. 2). Histological examination confirmed extensive benign angiokeratoma (Fig. 3). The patient made an excellent recovery with resolution of symptoms and restoration of sexual function, with a satisfactory cosmetic result recorded at 3 and 6 months postoperatively (Fig. 4).

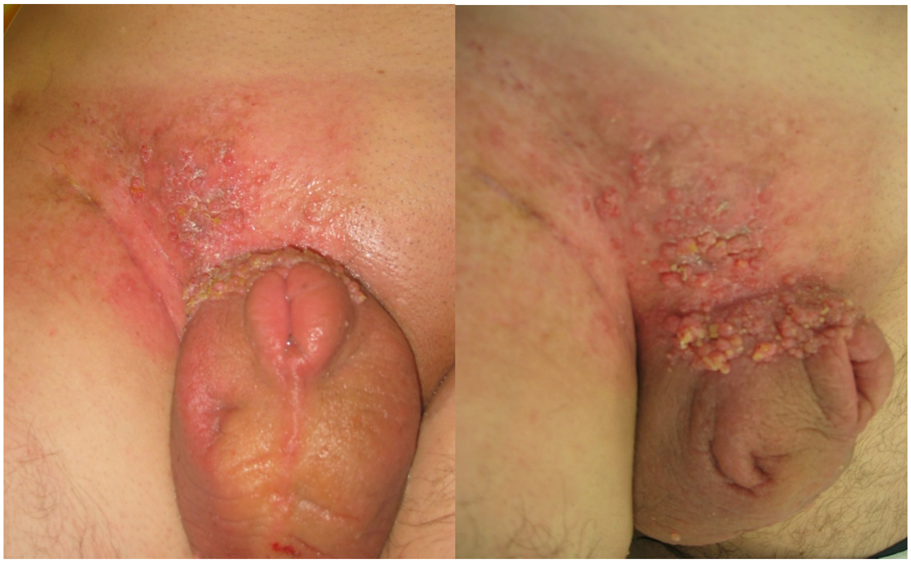

**FIGURE 1**. Extensive angiokeratoma of the right inguinal region, penile shaft, and scrotum in the distribution of the previous radiotherapy treatment. Also note the scrotal lymphoedema and buried penis.

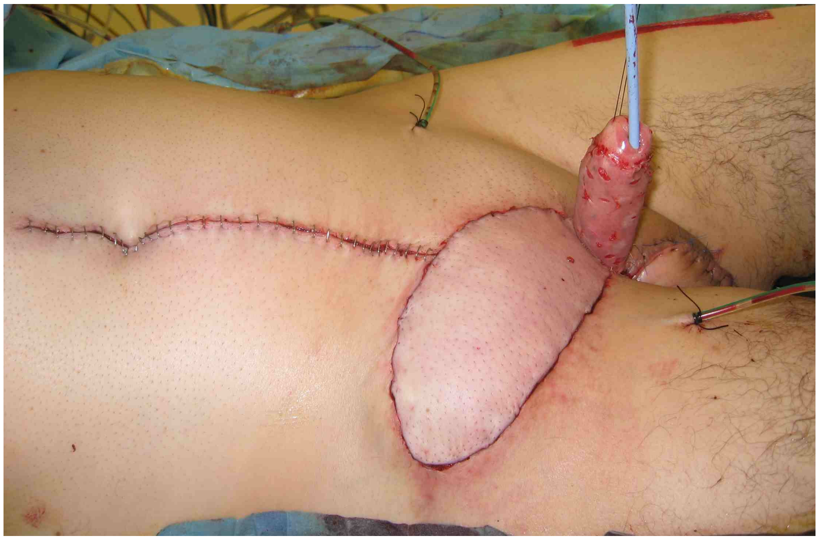

**FIGURE 2.** Replacement of diseased inguinal skin with a VRAM flap and penile shaft skin with a partial-thickness skin graft.

## DISCUSSION

Angiokeratomas are benign lesions thought to be telangectasias with secondary epithelial proliferation. They are histologically characterised by ectatic capillaries at the dermoepidermal junction, with reactive hyperkeratosis, acanthosis, and focal papillomatosis of the overlying epidermis[1]. A few angiokeratomas in the genital area are reasonably commonplace, but extensive areas are considered pathological. They have most commonly been described in the scrotum (Fordyce) and vulva[2], and are part of Fabry's disease, an X-linked lysosomal storage disorder. Suggested pathophysiological mechanisms include increased local venous pressure, injury to capillary walls, primary degeneration of the venous supportive elastic tissues, or chronic inflammation leading to phlebectasia[2].

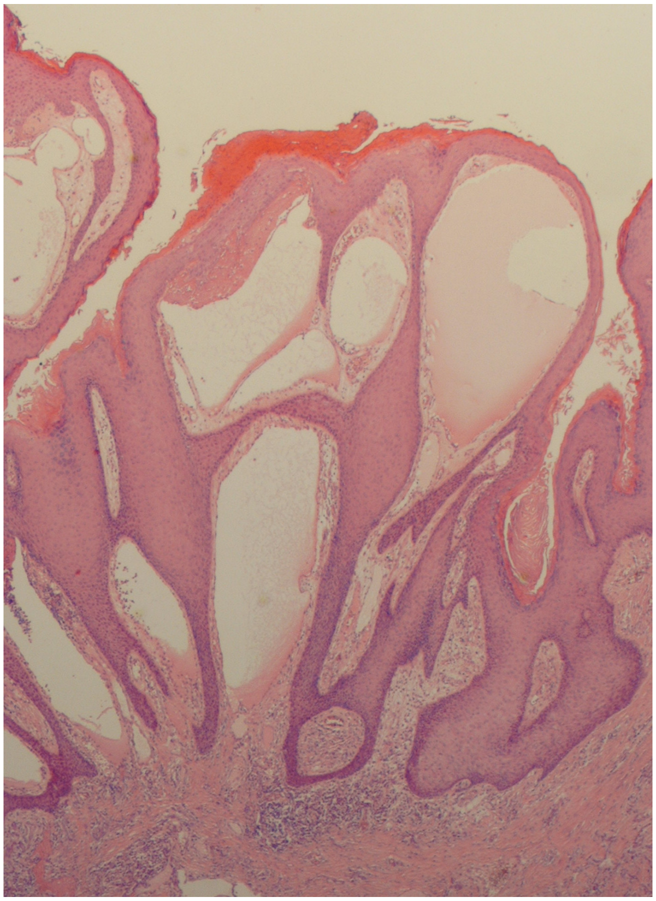

**FIGURE 3**. Examination of the pathological specimen shows collections of ectatic vessels at the dermoepidermal junction with acanthosis of the overlying epidermis, classical of benign angiokeratomas.

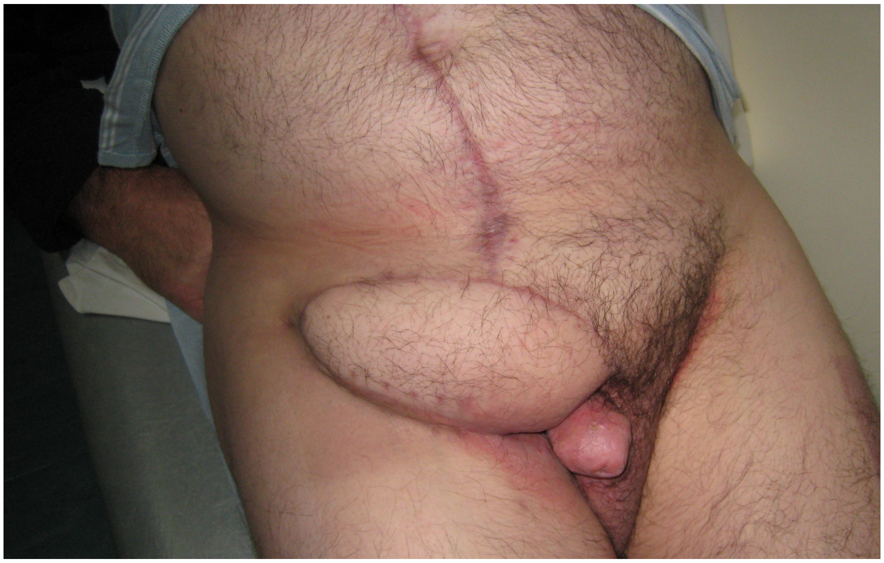

**FIGURE 4.** Cosmetic appearance at 3 months postoperatively. The patient resumed sexual activity 2 months later.

An association between radiotherapy and the development of angiokeratoma has been made in two reports following radiotherapy for vulval carcinoma[3,4], and one of the penile stump following penectomy and adjuvant radiotherapy for penile cancer[5]. A possible mechanism is vascular injury as a result of radiotherapy-induced free radical production. Where there has also been previous surgery, disruption of the venous anatomy may also be contributory. Given the previous history of malignancy, diagnosis is also an important factor in this context and biopsy is required to exclude disease recurrence.

Here we describe for the first time the development of extensive angiokeratoma of the groin, penile shaft, and scrotum following radiotherapy for lymph node–positive penile cancer. Our patient was particularly troubled by symptoms of pain and discharge, possibly due to the associated lymphoedema resulting from the previous lymphadenectomy and groin radiotherapy.

The treatment of angiokeratomas is not well documented, but is based on degree of symptoms (e.g., bleeding, pain, discharge) or cosmetic concerns. Reassurance may be all that is required. Local excision, cryotherapy, and electrocautery have been used for smaller lesions. Larger lesions may be treated with wide excision and the effective use of laser ablation has been described[5], although this can result in scarring.

Here we report an effective surgical solution for an extensive area of angiokeratoma involving the genital area. If such a large area is affected and symptomatic, wide excision with grafting and rotational flap is a viable and excellent option to offer complete resolution of symptoms.

## References

1. Atherton, D.J. and Moss, C. (2004) Naevi and other developmental defects. In *Rook's Textbook of Dermatology.* Burns, T., Breathnach, S., Cox, N., et al., Eds. Blackwell Science, Oxford. pp 15.87–15.90.
2. Cohen, P.R., Young, A.W., Jr., and Rovell, H.M. (1989) Angiokeratoma of the vulva: diagnosis and review of the literature. *Obstet. Gynecol. Surv*. **44,** 339–346.
3. Haidopoulos, D.A., Rodalakis, A.J., Elsheikh, A.H., et al. (2002) Vulval angiokeratoma following radical hysterectomy and radiotherapy. *Acta Obstet. Gynecol. Scand*. **81,** 466–467.
4. Smith, B.L., Chu, P., and Weinberg, J.M. (2004) Angiokeratomas of the vulva: possible association with radiotherapy. *Skinmed* **3(3),** 171–172.
5. Malalsekera, A.P., Goddard, J.C., and Terry, T.R. (2007) Angioleratoma of Fordyce simulating recurrent penile cancer. *Urology* **69,** 576.e13–576.e14.
